# Notes and News

**Published:** 1903-10-31

**Authors:** 


### Notes and News.

The King will lay the foundation stone of his Sanatorium at Lord's Common, Midhurst, Sussex, on November 3rd. The Sanatorium will be named King Edward VII. Sanatorium.

The Queen has sent £1,000 to the Lord Mayor for the new buildings and rebuilding of St. Bartholomew's Hospital.

Princess Henry of Battenberg will unveil on November 28th the memorial tablet to the late Emperor and Empress Frederick at the new Hospital for Women. The memorial takes the form of an endowment for a cancer bed in the hospital.

The proposed additions and improvements in the new buildings at University College of South Wales and Monmouthshire include departments for science, physics, chemistry, physiology, medicine, and public health. The public health department will co-operate with Cardiff and Glamorgan, which commenced work in temporary premises some years ago. A research laboratory will form part of the scheme, and should prove especially useful.

The scrutiny to which the health of our youthful population is now being subject, cannot fail to effectively improve the condition of the adult of the future. In most of the large cities the importance of observation of and attention to minor ailments in school children is becoming recognised, and efforts are being made to improve the lot of the bodily and mentally afflicted. Londoners are already becoming accustomed to the sight of the comfortable carriages which convey the crippled children between their homes and the schools, which, thanks largely to the energy of Mrs. Humphry Ward, now form part of the educational system of the London School Board. By means of these cripple schools not only are the mental capacities of the afflicted children developed so as to fit them to support themselves, but their physical condition is greatly improved by healthy environment and wholesome food.

Dr. R. Prosser White has presented a Finsen light apparatus to the Royal Albert Edward Infirmary, Wigan.

From Melbourne comes the report of the terrible experience of a steamship, when journeying from Penang to Singapore and Hong Kong, when they lost 263 Chinese coolies from cholera and the plague.

In Paris the manager of a French restaurant has obtained £200 compensation from a chemist who constantly sold morphia to his wife. The morphia habit has gained such a hold in Paris that the authorities are adopting severe repressive remedies.

The annual meeting of the presidents of the League of Mercy will be held in the board-room of the Charing Cross Hospital, by kind permission of the president and council of the hospital, on the afternoon of Friday, December 18th. Mr. E. A. Hambro, D.L., J.P., president of the Sevenoaks district and chairman for the year, will preside.

The Anti-Vivisectionists are trying to interfere with the Birmingham University by protesting against the granting of a license to conduct a laboratory for vivisection. They tried to enlist Mr. Chamberlain's sympathy, but he replied by expressing his approval of the application for the license on behalf of the University and his confidence in the well-known scientist who would be responsible.

At a meeting of the Westminster City Council last week, Colonel Probyn, who at the recent meeting at King's College Hospital, had strongly opposed the proposals to remove the hospital from its present site, again made a strong effort to gain supporters by pointing out that the removal was an injustice to Westminster. Although a motion to restrain was seconded, the meeting decided that the action of the hospital authorities should not be interfered with, on the grounds that Westminster was better supplied with hospitals than any other part of London.

The Bishop of Zanzibar, who himself is a medical man, writes that after completing a visit of some duration in the Magila Archdeaconry, he is convinced there is an opening and a need for a doctor in that part of the country. He reports that he has been asked to perform a greater number of operations than he could possibly undertake. The people like to come for treatment to the mission or to the mission dispensaries, and, with the present staff of nurses, the Bishop thinks there ought to be no difficulty in a competent man undertaking cases of the gravest nature. The doctor would find plenty to do, and possibly a good deal to investigate of scientific interest. He should have to some degree the missionary vocation, though he would not be required to do anything else except to pursue his own particular calling. "There is also the health of the European staff to be considered; that, too, requires a resident doctor." Dr. Oswald A. Browne, Member of the Medical Board of the Universities Mission to Central Africa, would be glad to give any further information at 9 Dartmouth Street, Westminster, S.W.

